# Effects of the mGluR5 antagonist MPEP on ethanol withdrawal induced anxiety-like syndrome in rats

**DOI:** 10.1186/1744-9081-9-43

**Published:** 2013-11-26

**Authors:** Jaya Kumar, Hermizi Hapidin, Yvonne-Tee Get Bee, Zalina Ismail

**Affiliations:** 1 BRAINetwork Centre for Neurocognitive Science, School of Health Sciences, Health Campus, Universiti Sains Malaysia, Kubang Kerian, Kelantan 16150, Malaysia; 2 School of Health Sciences, Health Campus, Universiti Sains Malaysia, Kubang Kerian, Kelantan 16150, Malaysia

**Keywords:** Ethanol withdrawal induced anxiety, MPEP, mGluR5

## Abstract

Abstinence from chronic ethanol consumption leads to the manifestation of a variety of symptoms attributed to central nervous system hyperexcitability, such as increased irritability, anxiety, and restlessness. Recent studies have demonstrated the importance of metabotropic glutamate receptor 5 (mGluR5) in addictive behaviours. This study investigates the effects of the mGluR5 antagonist 2-methyl-6-(phenylethynyl)-pyridine (MPEP) on ethanol withdrawal induced anxiety using two behavioural paradigms. Male Wistar rats were fed a Modified Liquid Diet (MLD) containing low fat cow milk, sucrose, and maltodextrin with a gradual introduction of 2.4%, 4.8% and 7.2% ethanol for 20 days. Six hours into ethanol withdrawal, the rats were intraperitoneally injected with normal saline and MPEP (2.5, 5.0, 10, 20, 30 mg/kg) and were assessed for ethanol withdrawal induced anxiety-like syndrome using an automated elevated plus maze and an open field. MPEP at 10 mg/kg significantly attenuated ethanol withdrawal induced anxiety without any compromising effects on locomotor activities. Despite reversing several indices of ethanol withdrawal induced anxiety in both the elevated plus maze and the open field, low doses of MPEP (2.5, 5 mg/kg) significantly compromised the locomotor activities of ethanol withdrawn rats. High doses of MPEP (20 and 30 mg/kg) significantly attenuated withdrawal anxiety when tested in the elevated plus maze but not in the open field. Administration of MPEP (2.5, 5, 10, 20, 30 mg/kg) has no significant compromising effect on the locomotor activities of ethanol naïve rats. Despite significantly reducing withdrawal anxiety in both behavioural paradigms at 10 mg/kg, the compromising effects of low and high doses of MPEP must be further explored along with the therapeutic efficiency of this drug for relieving withdrawal induced anxiety.

## Introduction

Glutamate is the most abundant excitatory neurotransmitter in the mammalian brain. The excitatory functions of glutamate are categorised into two types, fast and slow. The fast excitatory actions of glutamate are mediated by ionotropic *N*-methyl-D-aspartate (NMDA), α-amino-3-hydroxy-5-methyl-ioxyzole-4-propionicacid (AMPA), and kainate (KA) receptors. The slow glutamate responses are mediated by metabotropic glutamate receptors (mGluRs) through G-protein coupling with numerous intracellular signalling cascades that can modulate ionotropic receptor function [1]. According to sequence homology, effector coupling, and pharmacology, mGluRs are divided into three subgroups. The group I mGluRs, metabotropic glutamate receptor 1 (mGluR1) and metabotropic glutamate receptor 5 (mGluR5), are positively coupled to phospholipase C and the group II mGlu receptors (mGlu2and mGlu3), and the group III receptors (mGlu4, mGlu6, mGlu7, and mGlu8) are negatively coupled to adenylate cyclase [2]. Among these mGluRs, mGluR5 has been shown to play an important role in ethanol seeking and relapse-like behaviours [3]. Additionally, 6-methyl-2-(phenylethynyl)-pyridine (MPEP), a selective mGluR5 antagonist, has been shown to reduce ethanol consumption [4], inhibit the onset and maintenance of ethanol self-administration [5], and reduce binge ethanol intake in the drinking in the dark paradigm [6].

MPEP also has been shown to possess anxiolytic property using several models of anxiety [7-9]. This present study addresses the importance of mGluR5 in both ethanol dependence and anxiety by exploring the role of mGluR5 in ethanol withdrawal induced anxiety. The anxiety-like syndrome that appears during abstinence from chronic ethanol exposure is an unpleasant feeling or negative emotional response accompanied by an increased glutamatergic neurotransmission [10]. This anxiety-like syndrome can contribute to an enhanced risk of relapse [11,12]. A limited number of studies has implicated the importance of mGluR5 in the manifestation of ethanol withdrawal induced anxiety-like syndrome [13]. Acamprosate, an FDA approved drug for the treatment of alcohol use disorders, has been shown to alter glutamatergic neurotransmission via a weak antagonism of NMDA receptors [14] and by an indirect blockade of the mGlu5 receptor [15]. The present study used MPEP, which is a selective mGluR5 antagonist of the human mGluR5a receptor with an IC 50 value of 36 nM, in a PI hydrolysis assay without any significant effect at other metabotropic or ionotropic glutamate receptors. MPEP has not shown agonist activity on group II mGluRs and readily penetrates the blood–brain barrier [16].

To investigate the role of mGluR5 in the manifestation of ethanol withdrawal induced-anxiety like syndrome, rats were exposed to chronic ethanol for 20 days using a Modified Liquid Diet (MLD) containing ethanol to create alcohol dependence. The rats were subsequently injected with respective doses of intraperitoneal (IP) saline and MPEP (2.5, 5.0, 10, 20, 30 mg/kg i.p) after six hours of withdrawal. Next, the rats were tested for ethanol withdrawal induced anxiety using an automated elevated plus maze system and an open field.

## Methods

### Animal preparation

All experiments were performed using male Wistar rats weighing 250-300 g obtained from the Laboratory Animal Research Unit, Universiti Sains Malaysia (LARUSM) and were maintained in a 12 h light–dark cycle with the lights on between 1900–0700. The animals were housed individually and kept at a constant room temperature of 24°C and were allowed to adapt to the surroundings for at least 7 days prior to the experiment. All of the animal procedures in this study were approved by the Animal Ethics Committee of Universiti Sains Malaysia.

The animals were allocated into 7 groups for each study (n = 56). Group 1: The rats were given MLD without ethanol (n = 8). Group 2 (n = 8) consisted of rats that were fed MLD with ethanol and were given an injection (i.p.) of normal saline 6 hours after the last ethanol intake. Group 3, 4, 5, 6, and 7 (n = 8/group) consisted of rats that received MLD with ethanol and received MPEP (2.5, 5, 10, 20 and 30 mg/kg, respectively) during withdrawal.

The rats were individually housed and were fed with an MLD without ethanol for 7 days in special glass bottles to prevent spillage. Group 1 was given an MLD without ethanol throughout the experiment. The rats had access to MLD for 24 hours a day. The MLD was prepared fresh and given in spillage free special drinking bottles twice per day as a sole diet at 0900 and 1900. At the end of 7 days, ethanol was gradually introduced into the MLD for groups 2, 3, 4, 5, 6, and 7 from 2.4% (3 days) to 4.8% (3 days) and 7.2% (14 days). When the ethanol concentration was increased, the sucrose and maltodextrin was reduced to maintain isocaloricity of the diet. The daily ethanol intake was measured twice per day at the same time for all rats and was expressed as grams per kilogram per day. After 20 days of chronic ethanol consumption, the ethanol was removed from the MLD and was replaced with sucrose and maltodextrin.

The effects of MPEP alone on anxiety and locomotor activity in the open field was carried out by measuring the time spent in the central zone and number of lines crossed in the open field by ethanol naive rats. Similarly, the effect of MPEP alone on anxiety (percent open arm total time and entries) and locomotor activities (basic movement, fine movement, X ambulation, Y ambulation) in the EPM was assessed for 5 minutes following MPEP administration (2.5, 5, 10, 20, and 30 mg/kg). Approximately 6 hours after the last ethanol intake and one hour before the behavioural testing, ethanol withdrawn rats were administered with MPEP (i.p.) and were brought to the behavioural study room. Each rat was handled carefully and consistently to minimise any sort of stress prior to behaviour testing.

### Elevated plus maze

The automated maze (Kinder Scientific, Poway, CA) consisted of two open arms (width, 10.8 cm, length, 50.17 cm) and two closed arms (width, 10.8 cm, length, 50.17 cm, walls, 40.01 cm) with a central platform (10.8cmx10. 8 cm). The maze was elevated 85.09 cm from the floor, and the rat movements were tracked by infrared photobeams embedded along the entire length of the base of each arm. The movements were subsequently analysed by Motor Monitor computer software. The locomotion of ethanol dependent and non-dependent rats in the maze was measured using an ambulation (a measure used to express larger animal movements) algorithm. The automated elevated plus maze was equipped with one Anchor Beam for each dimension, one X Anchor Beam and one Y Anchor Beam. The Anchor Beam is the lowest beam blocked in a dimension. The Anchor Beam is reset when an animal ambulates. An ambulation occurs when a new beam block occurs, and the anchor beam for that dimension is released before the new beam. For example, if *X*2, X3, X4 are blocked and then X5 is blocked, the new beam break at X5 will be counted as an ambulation. Otherwise, the break at X5 is recorded as a fine movement. Fine movement is used to express smaller animal movements, such as grooming and head movements. Fine movement is recorded when a subject changes a beam status but the change does not fit the definition of an ambulation. The fine movement counter is incremented when the beam status change does not meet the ambulation algorithm. Basic movement is the simple tally of all horizontal beams in the system. The basic movement counter is incremented upon each new beam block. The experiments were conducted during the dark phase of a light–dark cycle in a quiet room with homogenous illumination (2–4 lx) directed towards the apparatus [17]. The experiment was initiated by placing the rat in the centre of the maze platform facing an open arm and was followed by recording the activity of the rat in the maze for 5 minutes of a single session for each rat. The maze was wiped clean after each test session. The ethanol withdrawal induced anxiety was measured as the time spent in open arms as a percent of the total time spent exploring both the open and closed arms (Open Arms Total Time Percentage) and the number of entries into the open arms as a percentage of the total number of entries into both open and closed arms (Open Arms Entries Percentage). The effects of MPEP alone on anxiety was measured by measuring the percent open arm total time and entries produced by ethanol naive rats.

### Open field test

The open field consisted of a square box that measured 60x60cm with 35 cm walls. Lines were drawn on the floor into 15x15cm squares and were visible through the clear Plexiglas floor. The test arena was divided into central and peripheral zones. Each rat was placed in the central area and was allowed to explore for 5 minutes. After the 5 minute test, the rats were returned to the home cages, and the open field was cleaned thoroughly and allowed to dry between tests. The apparatus was placed under a homogenous illumination (14–20 lx) [17]. The performance in the open field was scored by video. The number of lines crossed by the ethanol dependent and non-dependent rats for 5 minutes in the open field was recorded as a measurement for locomotor activities where all 4 paws required crossing a line for a count to occur. Ethanol withdrawal induced anxiety was recorded by measuring the percentage of time spent in the central zone and the number of entries produced in the central zone of the open field. The effects of MPEP alone on anxiety was assessed by measuring the percent total time spent in the central zone of the open field by ethanol naive rats following MPEP treatment.

### Drugs

The mGluR5 antagonist MPEP (2-methyl-6-(phenylethynyl)-pyridine) was purchased from Tocris, UK. The drug was freshly dissolved in physiological saline, and injected i.p. MPEP was administered in the doses of 2.5, 5, 10, 20 or 30 mg/kg (2 ml/kg). Ethanol was purchased from Hamburg Chemicals, and ethanol stock solutions were prepared by mixing appropriate volumes of ethanol (95.6% v/v) and distilled water. The dose range of the drug and the time of administration were chosen based upon [18]. The dose 2.5 mg/kg MPEP was chosen for ethanol naive rats based on a previous study [9] and our preliminary study.

### Modified liquid diet (MLD)

The composition of the MLD with ethanol was low fat cow milk (12%), ethanol 95.6% (2.4, 4.8, and 7.2% of the solution), maltodextrin (10.35%) and 17 g sucrose. The final volume of the MLD was maintained at 1 L. MLD without ethanol was isocaloric to MLD with ethanol and contained sucrose and maltodextrin as a caloric substitute for ethanol. This mixture supplied 1070 kcal l^-1^ and was a modification of the MLD proposed by Uzbay and Kayaalp [19].

### Blood ethanol level measurement

#### Tail blood collection

Tail blood samples (0.5 ml) were taken from a separate individual group of rats (n = 20) that were fed MLD containing ethanol for 20 days. The blood sample was taken immediately after removing ethanol from the liquid diet [19] and were centrifuged to obtain the sera. The sera were sent to the Doping Control Centre, USM Penang to measure the blood ethanol levels. The blood ethanol levels were measured using a Gas Chromatograph(y) Flame Ionisation Detector (GC FID).

#### Chemicals and reagents (blood ethanol level measurement)

Deionised water (18 MΩ cm resistivity) was obtained from Elga Purelab water purification system (ELGA, UK). Alcohol (Ethyl Alcohol with purity 99.4%) was supplied by Fisher Scientific, and 1-propanol (Internal Standard), purity 99.5%, was obtained from Merck.

#### Instruments

The 20 mL headspace vials were incubated in a headspace autosampler (Agilent G1888 headspace Sampler) at 70°C for 15 min. After equilibration, 0.2 ml of the headspace vial was pressurised into the GC/FID. The loop and transfer line temperatures were set at 75°C and 80°C, respectively. Alcohol analysis was performed with an Agilent Flame ionisation detector (FID) (Agilent, USA) equipped with Agilent GC 6890 series (Agilent, USA). The column used was an Agilent HP5 fused silica capillary column (30 m x 0.32 mm, film thickness 0.32 um). The injector and detector temperatures were 200°C and 250°C, respectively. Hydrogen (40 mL/min) was the carrier gas. The oven temperature was set at isothermal 50°C for 5 min. The retention time for alcohol was 3.17 min, and the retention time for 1-propanol (Internal Standard) was 3.25 min. Good linearity (r2 > 0.99) was obtained, and the blood ethanol assay was performed in triplicate of each sample [20].

### Statistical analysis

Data were expressed as the mean ± SEM. Data for the final average ethanol intake, elevated plus maze study and open field test were analysed by one way analysis of variance (ANOVA), and the differences between individual means were compared with a post hoc Tukey’s Test. p < 0.05 was considered to be statistically significant.

## Results

### Ethanol consumption and blood ethanol level

The average ethanol intake for 20 days ranged from 10.1 ± 0.7 to 10.9 ± 0.4 g/kg^-1^ day^-1^ (Table 1). There were no significant differences in the amount of ethanol consumed between groups [F(6,49) = 0.357; p > 0.05]. The blood ethanol level of the ethanol fed rats was 283.1 ± 14.5mgdl^-1^(n = 20) just before abstinence.

**Table 1:** Average ethanol intake

** Group**	**Ethanol intake(g/kg day**^**-1**^**)**
Ethanol Withdrawal (EW)	10.2 ± .5
EW + 2.5MPEP	10.5 ± .4
EW + 5MPEP	10.9 ± .4
EW + 10MPEP	10.2 ± .4
EW + 20MPEP	10.4 ± .2
EW + 30MPEP	10.1 ± .7

Table 1 shows average ethanol intake (20 days) of the animals employed in the behavioral study, One Way Analysis of Variance.

### Basic movement

**Figure 1:**
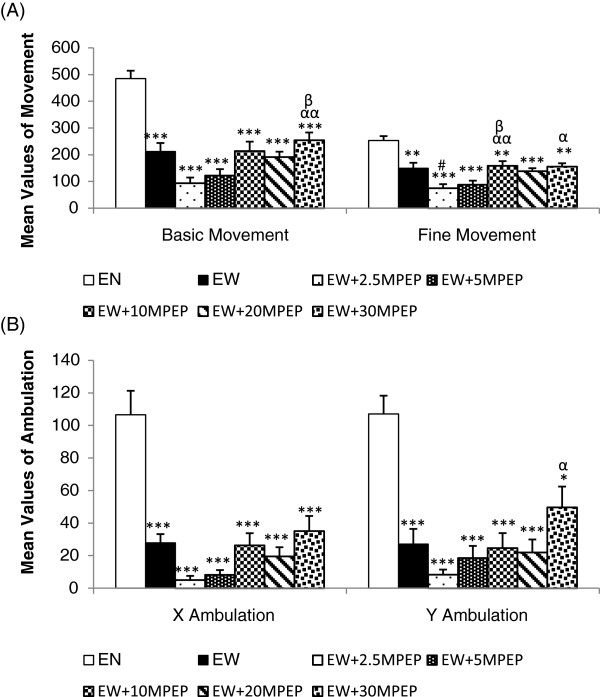
**The effect of MPEP on the locomotion of ethanol withdrawn rats.** The effect of MPEP (2.5, 5, 10, 20, and 30 mg/kg) on basic and fine movement **(A)**, X and Y ambulation **(B)** in the automated elevated plus maze of ethanol withdrawn rats after 7 hours of withdrawal. Each column represents the mean ± S.E.M [n = 8 for each group; EN = Control rats fed MLD without ethanol and treated with saline; EW = Ethanol Withdrawal (the ethanol withdrawn group treated with normal saline); EW + 2.5, EW + 5,EW + 10, EW + 20, EW + 30 MPEP = ethanol withdrawn group treated with respective doses of MPEP (mg/kg); **p < 0.01,***p < 0.001 vs EN; #p < 0.05 vs EW; αp < 0.05, ααp < 0.001 vs 2.5 mg/kg MPEP; βp < 0.05 vs 5 mg/kg MPEP, One Way Analysis of Variance and post hoc Tukey’s test].

The basic movement was recorded by monitoring rat behaviour in the automated elevated plus maze. One Way Analysis of Variance (ANOVA) revealed a significant reduction in the basic movement of the ethanol fed rats compared to control rats [F(6,49) = 20.975; p < 0.0001] (Figure 1A). Administration of 2.5 and 5 mg/kg MPEP resulted in a significant reduction in the basic movement compared to dose of 30 mg/kg MPEP. However, there were no significant differences recorded between the vehicle and MPEP (10, 20, 30 mg/kg) treated ethanol fed rats (Figure 1A).

### Fine movement

Fine movement was obtained by assessing the activity of the rats in the automated elevated plus maze. A significant reduction in the fine movement of ethanol fed rats was observed compared to the control rats [F(6,49) = 13.548; p < 0.0001] (Figure 1A). Administration of 2.5 mg/kg MPEP resulted in a significant reduction in fine movement compared to ethanol withdrawal and MPEP at doses of 10 and 30 mg/kg. Significant differences in fine movement were recorded between animals given 5 mg/kg MPEP and 10 mg/kg MPEP. However, there were no significant differences between the vehicle and MPEP (10, 20, 30 mg/kg) treated ethanol fed rats (Figure 1A).

### X and Y ambulation

Similar to both basic and fine movements, a significant reduction in X ambulation (closed arm) of ethanol fed rats was observed compared to control rats [F(6,49) = 19.456; p < 0.0001] (Figure 1B). There were no significant differences between the ethanol withdrawn group and the MPEP (2.5, 5, 10, 20, 30 mg/kg) treated rats (Figure 1B). Likewise, Y ambulation (open arm) of ethanol withdrawn group is significantly lower than the Y ambulation of the control group [F(6,49) = 13.173; p < 0.01] (Figure 1B).

### Open Arm total time (%)

**Figure 2:**
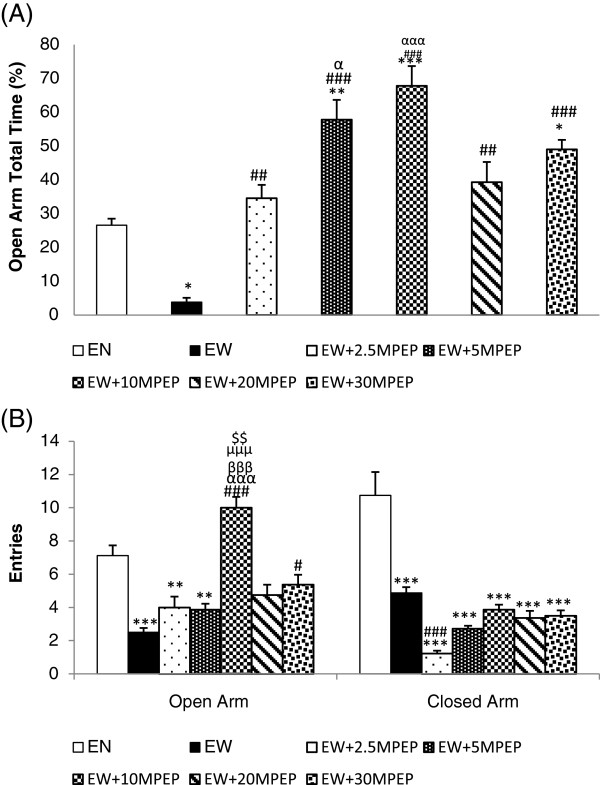
**The effect of MPEP on open and closed arm behaviours.** The effect of MPEP (2.5, 5, 10, 20, and 30 mg/kg) on percent total time spent in the open arms **(A)**, and the open and closed arm entries **(B)**of the automated elevated plus maze of ethanol withdrawn rats 7 hours after withdrawal. Each column represents the mean ± S.E.M [n = 8 for each group; EN = Control rats fed MLD without ethanol and treated with saline; EW = Ethanol Withdrawal (ethanol withdrawn group treated with normal saline); EW + 2.5, EW + 5,EW + 10, EW + 20, EW + 30 MPEP = ethanol withdrawn group treated with respective doses of MPEP (mg/kg);*p < 0.05,**P < 0.01, ***p < 0.001 vs EN; #p < 0.05, ##p < 0.01,###p < 0.001 vs EW; αp < 0.05, αααp < 0.001 vs 2.5 mg/kg MPEP; βββp < 0.001 vs 5 mg/kg MPEP; μμμp < 0.001 vs 20 mg/kg MPEP; $$p < 0.01 vs 30 mg/kg MPEP,One Way Analysis of Variance and post hoc Tukey’s test].

Figure 2A illustrates a significant decrease in the total time spent in the open arms of the elevated plus maze in the ethanol withdrawn rats compared to rats fed MLD without ethanol [F(6,49) = 20.309; p < 0.0001]. The post Hoc analysis revealed a significant increase in the time spent in the open arms compared to the ethanol withdrawal group following administration of 2.5, 5, 10, 20, and 30 mg/kg MPEP. During ethanol withdrawal, rats treated with 5 and 10 mg/kg of MPEP spent significantly more time in the open arms of the elevated plus maze than rats treated with 2.5 mg/kg MPEP (Figure 2A).

### Open Arm entries

Similar to the open arm total time percentage, a decrease in the entries in the open arms of the elevated plus maze was reported in ethanol withdrawn rats compared to rats fed MLD without ethanol [F(6,49) = 15.565; p < 0.0001] (Figure 2B). Post hoc analysis revealed a significant increase in the open arm entries following the administration of 10, and 30 mg/kg MPEP compared to the ethanol withdrawn group. During abstinence, rats treated with 10 mg/kg MPEP produced significantly more entries in the open arm of the maze compared to animals given 2.5, 5, 20 and 30 mg/kg MPEP.

### Closed Arm entries

Figure 2B shows a decrease in the closed arm entries of the elevated plus maze in ethanol withdrawn rats compared to rats fed MLD without ethanol [F(6,49) = 41.044; p < 0.0001]. Administration of 2.5 mg/kg MPEP resulted in a significant decrease in the closed arm entries compared to ethanol withdrawal group shown by post hoc analysis. However, there were no significant changes in the closed arm entries recorded between vehicle and MPEP (5, 10, 20, 30 mg/kg) treated ethanol withdrawn rats (Figure 2B).

### Basic and fine movement of ethanol naïve rats

**Figure 3:**
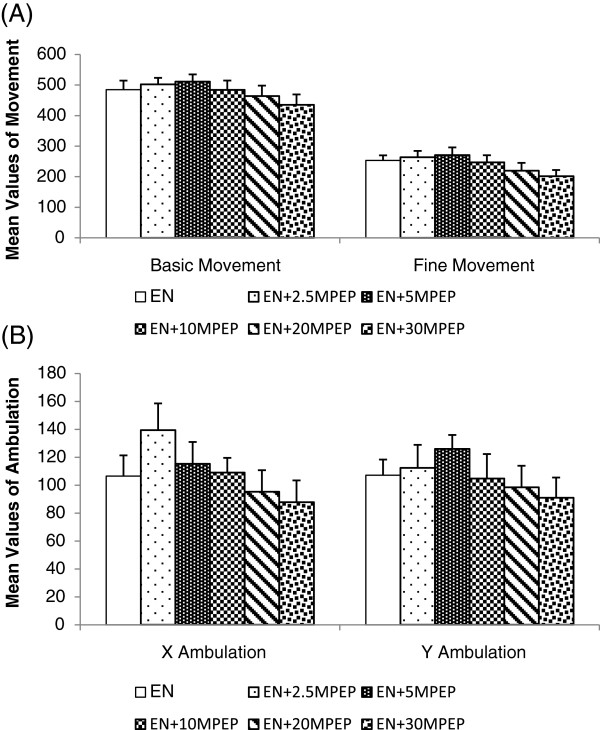
**The effect of MPEP on locomotion of ethanol naïve rats.** The effect of MPEP (2.5, 5, 10, 20, and 30 mg/kg) on basic and fine movement **(A)**, X and Y ambulation **(B)** in automated elevated plus maze of ethanol naïve rats. Each column represents the mean ± S.E.M [n = 8 for each group; EN = Control rats fed MLD without ethanol and treated with saline; EN + 2.5,EN + 5,EN + 10, EN + 20, EN + 30MPEP = ethanol naive group treated with respective doses of MPEP (mg/kg); One Way Analysis of Variance].

Figure 3A shows the effect of MPEP (2.5, 5, 10, 20, 30 mg/kg) on the basic movement of ethanol naïve rats. Neither doses of MPEP produced any significant effect on the basic movement of the ethanol naïve rats [F(5,42) = 0.892; p > 0.05] (Figure 3A). Likewise, MPEP (2.5, 5, 10, 20, 30 mg/kg) had no significant effect on the fine movement of ethanol naïve rats [F(5,42) = 1.767; p > 0.05] (Figure 3A).

### X and Y ambulation of ethanol naïve rats

**Figure 4:**
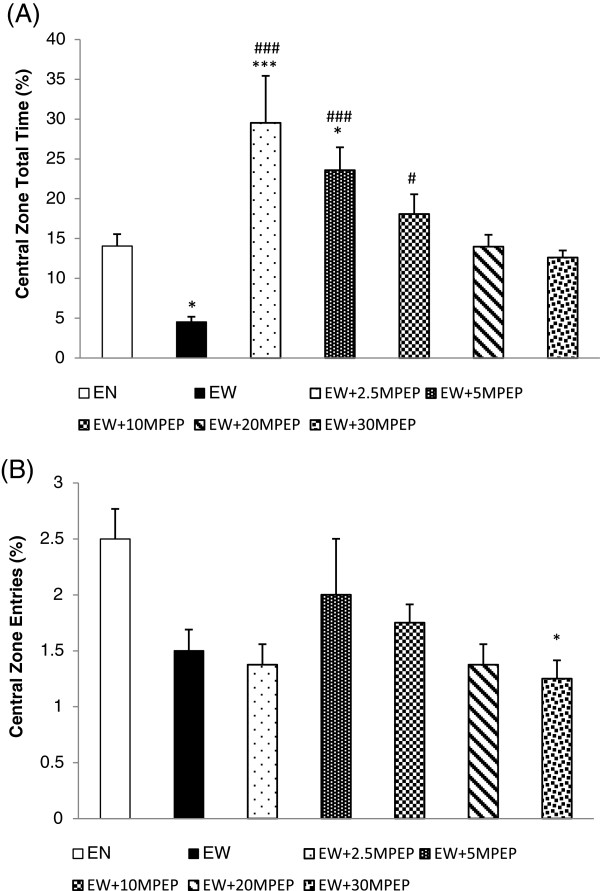
**The effect of MPEP on open field behaviours.** The effect of MPEP (2.5, 5, 10, 20, and 30 mg/kg) on the percent total time **(A)** and the entries **(B)** produced in the central zone of the open field by ethanol withdrawn rats 7 hours after withdrawal. Each column represents the mean ± S.E.M [n = 8 for each group; EN = Control rats fed MLD without ethanol and treated with saline; EW = Ethanol Withdrawal (ethanol withdrawn group treated with normal saline); EW + 2.5, EW + 5,EW + 10, EW + 20, EW + 30MPEP = ethanol withdrawn group treated with respective doses of MPEP (mg/kg); *p < 0.05, ***p < 0.001 vs EN; #p < 0.05, ###p < 0.001 vs EW, One Way Analysis of Variance and post hoc Tukey’s test].

Figure 3B shows the effect of MPEP (2.5, 5, 10, 20, 30 mg/kg) on the X ambulation of the ethanol naïve rats. Neither doses of MPEP produced any significant effects on the X ambulation of ethanol naïve rats [F(5,42) = 1.936; p > 0.05] (Figure 4A). Similarly, MPEP (2.5, 5, 10, 20, 30 mg/kg) had no significant effect on the Y ambulation of the ethanol naïve rats [F(5,42) = 2.312; p > 0.05] (Figure 3B).

### Central zone total time (%)

The effect of MPEP (2.5, 5, 10, 20, and 30 mg/kg) on the percentage of total time spent in the central zone of the open field in ethanol withdrawn rats is shown in Figure 4A. One Way Analysis of Variance (ANOVA) reveals a significant decrease in total time spent in the central zone of the open field in the ethanol withdrawn rats compared to rats fed MLD without ethanol [F(6,49) = 13.995; p < 0.0001] (Figure 4A). Post Hoc analysis revealed a significant increase in the time spent in the central zone compared to ethanol withdrawal group following administration of 2.5, 5, and 10 mg/kg MPEP. However, treatment of ethanol withdrawn rats with MPEP (20, 30 mg/kg) did not significantly affect the time spent in the central zone of the open field.

### Central zone entries (%)

As seen in Figure 4B, the ethanol withdrawn rats exhibited a decrease in the entries in the central zone of the open field (statistically insignificant) compared to the ethanol naïve rats [F(6,49) = 2.889; p < 0.05]. No dose of MPEP (2.5, 5, 10, 20, 30 mg/kg) significantly affected the percentage of central zone entries in the open field. However, post hoc analysis revealed that administration of 30 mg/kg MPEP resulted in a significant reduction in central zone entries compared to normal rats.

### Open Arm total time and entries in ethanol naive rats(%)

**Figure 5:**
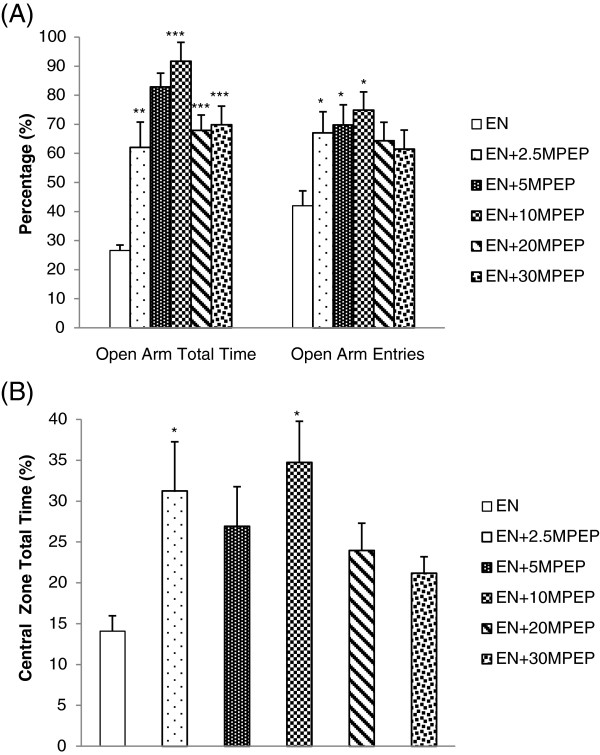
**The effect of MPEP on the anxiety of ethanol naïve rats in the automated elevated plus maze and open field.** The effect of MPEP (2.5, 5, 10, 20, and 30 mg/kg) on the percent open arm total time and entries of the automated elevated plus maze **(A)** and percentage of total time spent in the central zone of the open field by ethanol naïve rats **(B)**. Each column represents the mean ± S.E.M [n = 8 for each group; EN = Control rats fed MLD without ethanol and treated with saline; EN + 2.5, EN + 5, EN + 10, EN + 20, EN + 30MPEP = ethanol naive group treated with respective doses of MPEP (mg/kg); *p<0.05, **p<0.01, ***p< 0.001 vs EN, One Way Analysis of Variance and post hoc Tukey’s test].

Figure 5A shows the effect of MPEP on percentage of open arm total time and entries in ethanol naive rats. MPEP (2.5, 5, 10, 20, 30 mg/kg) significantly increased the total time spent in the open arm of the maze [F(5,42) = 14.455; p < 0.001]. However, only rats administered MPEP (5, 10 mg/kg) significantly increased percentage the of open arm entries produced [F(5,42) = 3.079; p < 0.05].

### Central zone total time in ethanol naive rats (%)

Figure 5B exhibits the effects of MPEP on percentage of time time spent in central zone of the open field by ethanol naive rats. MPEP (2.5, 10 mg/kg) significantly increased the percentage of time spent in the central zone of the open field [F(5,42) = 3.073, p < 0.05).

### Lines crossed

**Figure 6:**
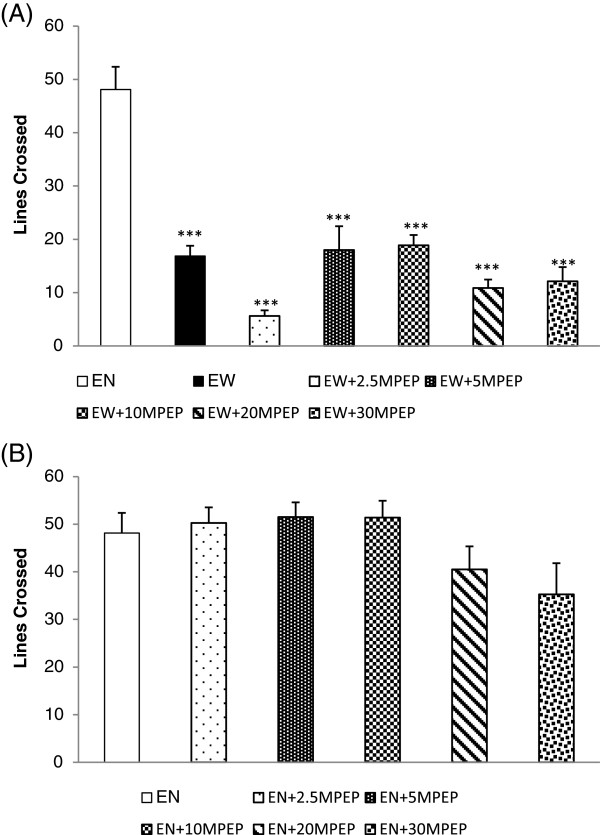
**The effect of MPEP on the number of lines crossed in open field.** The effect of MPEP (2.5, 5, 10, 20, and 30 mg/kg) on the number of lines crossed in the open field by ethanol withdrawn rats 7 hours after withdrawal **(A)** and ethanol naïve rats **(B)**. Each column represents the mean ± S.E.M [n = 8 for each group; **(A)** EN = Control rats fed MLD without ethanol and treated with saline; EW = Ethanol Withdrawal (ethanol withdrawn group treated with normal saline); EW + 2.5, EW + 5,EW + 10, EW + 20, EW + 30MPEP = ethanol withdrawn group treated with respective doses of MPEP (mg/kg); **(B)** EN + 2.5, EN + 5,EN + 10, EN + 20, EN + 30MPEP = ethanol naive group treated with respective doses of MPEP (mg/kg); ***p < 0.001 vs EN, One Way Analysis of Variance and post hoc Tukey’s test].

The effect of MPEP (2.5, 5, 10, 20, and 30 mg/kg) on number of lines crossed in the open field by ethanol withdrawn rats is shown in Figure 6A. One Way Analysis of Variance (ANOVA) revealed a significant decrease in the number of lines crossed in the open field by the ethanol withdrawn rats compared to rats fed MLD without ethanol [F(6,49) = 14.543; p < 0.001]. No dose of MPEP (2.5, 5, 10, 20, 30 mg/kg) had a significant effect on the number of lines crossed in the open field during ethanol withdrawal. Figure 6B shows the effect of MPEP (2.5, 5, 10, 20, 30 mg/kg) on the number of lines crossed by ethanol naïve rats in the open field. No significant difference was reported between the control and the MPEP treated ethanol naïve rats [F(5,42) = 2.43; p > 0.05].

## Discussion

The liquid diet technique was chosen as a method of ethanol administration in this study as this is the most comparable and relevant model for ethanol consumption in humans [19]. The lack of group differences in the average ethanol intake (Table 1) prior to drug administration negates the contribution of pre-existing differences in ethanol exposure to the dose dependent effects of the antagonist on anxiety. This current study demonstrates the anxiolytic property of MPEP using the anxiety models of the automated elevated plus maze and the open field test. Evaluation of animal behaviour in the elevated plus maze is based on two conflicting tendencies: the explorative drive of rodents in a novel environment and the aversion to open spaces. Anxious animals spend more time in the closed arms, while less anxious animals explore the open arms longer [21]. However, the anxiety-like behaviour in the open field is demonstrated through the conflict between exploration and the aversion against open, bright areas [22]. An important feature of rats in this anxiety model is traveling close to the wall, which confers security, while the centre is anxiogenic [23]. Anxious rodents spend more time in the corner and at the periphery of the arena as a natural tendency of avoidance reaction. A considerable amount of literature on ethanol withdrawal induced anxiety has been published using these models [24,25]. Both tests assess the unconditioned response to aversive environments; however, the findings of previous studies corroborate the idea that these tests measure different aspects of anxiety [26,27].

Our study demonstrates that withdrawal from ethanol following 20 days of chronic ethanol consumption induced an anxiety-like state in rats when tested in the automated elevated plus maze and the open field. This ethanol withdrawal induced anxiety was observed as a decrease in the percentage of open arm total time, the open arm entries, the central zone total time, the central zone entries, and the locomotion. Our study indicated that 10 mg/kg of MPEP (i.p) increased the open arm total time, the open arm entries, and central zone total time percentage in ethanol withdrawn rats. MPEP at 30 mg/kg produced a significant anxiolytic effect in ethanol withdrawn rats when tested in the automated elevated plus maze. This significant effect of MPEP in reducing anxiety at 10 mg/kg is similar to some previous findings [8,9]. These previous studies reported the anxiolytic properties of MPEP in normal rats, while our study is reporting the anxiolytic properties of MPEP using an ethanol withdrawal model for the first time. Our study recorded significant anxiolytic effects of MPEP at 2.5 mg/kg in ethanol naive rats. However, the significant anti-anxiety effect of MPEP in reversing withdrawal anxiety occurred at 10 and 30 mg/kg MPEP when tested in the elevated plus maze (both percent open arm total time and entries). These discrepancies in dose responses clearly indicate the possibilities of changes in mGluR5 expression or function during ethanol withdrawal as suggested previously by Olive and Becker [28]. Chronic exposure to an ethanol containing liquid diet decreased mGluR5 mRNA levels in the dentate gyrus and CA3 regions of the rat hippocampus [29]. Another study using a mature organotypic hippocampal slices demonstrated an increase in mGluR5 (not significant), and the NR1 and NR2B subunits of NMDARs following 10 days of ethanol exposure [30]. Another study by Obara and colleagues reported an increase in the expression of mGluR5 in the nucleus accumbens and the central amygdala of P (alcohol-preferring) rats following chronic ethanol consumption and withdrawal [31]. Acutely, ethanol inhibits the function of both NMDA and mGluRs [32,33]. Thus, chronic ethanol consumption results in a compensatory increase in the expression and the sensitisation of acutely inhibited glutamate receptors [34]. Contrary to previous studies where low doses of MPEP were sufficient to demonstrate significant anxiolytic property [8,9], this study demonstrates significant anti-anxiety effects of MPEP at moderate and high doses when tested in the elevated plus maze. Thus, the results of this study indirectly corroborate the findings of the previous works that demonstrated the upregulation of mGluR5 following chronic ethanol intake.

Another finding of this study was that MPEP at high doses had no significant effect on any indices of withdrawal anxiety when tested in the open field but showing significant anxiolytic effects in the automated elevated plus maze. MPEP at a dosage of 30 mg/kg was recommended by (Anderson et al., 2003) for a maximum effect, but no significant effect on ethanol withdrawal induced anxiety was recorded at either 20 or 30 mg/kg when tested in the open field. This contradicting treatment effect observed in different tests might be due to differences in the psychobiological meanings of various tests [35,36]. For example, chlordiazepoxide produced anti-anxiety effects in elevated plus maze but not in the open field in Lewis rats. This inter-test variation suggests that the construct differences between these tests assess different aspects of anxiety [26,27]. Thus, the variations in dose effects observed in this study might be due to the construct difference of these two behavioural paradigms. In addition, the possible off target effects of MPEP at high doses could influence the animal behaviours observed in this study. According to O’Leary et al. [37], MPEP is a non-competitive NMDA receptor antagonist and decreases NMDA or glutamate-induced neurotoxicity through NMDA antagonism. Another study demonstrated the functional interplay between mGluR5s and NMDARs during ethanol withdrawal induced neurotoxicity using organotypic hippocampal slices [38]. In addition, Olive [39] reported that the high dose effect of MPEP can be due to mechanisms not associated with mGluR5 modulation.

Any sedative or compromising effects on locomotor activities can produce confounding results in a behavioural study. We recorded the locomotion of ethanol withdrawn and ethanol naïve rats using multiple parameters, including basic movement, fine movement, X ambulation (closed arm), Y ambulation (open arm), closed arm entries, and the number of lines crossed in the open field. Our data shows that 20 days of chronic ethanol administration results in a significant reduction in locomotor activities of ethanol withdrawn rats compared to control rats (Figure 1 and 5A). Ethanol fed rats showed a significant reduction in basic and fine movement, X and Y ambulation, the number of entries produced in the closed arms of the elevated plus maze and the number of lines crossed in the open field compared to control rats. This ethanol withdrawal induced hypolocomotion following chronic ethanol exposure is synonymous with some previous studies [40,41] and withdrawal induced hypoactivity is an additional behavioural sign of ethanol withdrawal [12]. MPEP at 2.5 and 5 mg/kg significantly decreased basic movement, fine movement, X ambulation, and Y ambulation in the elevated plus maze. Additionally, neither doses of MPEP (2.5 and 5 mg/kg) imposed any significant effect on the open arm entries compared to the ethanol withdrawal group, suggesting that the significant anxiolytic effect of low doses of MPEP recorded in the elevated plus maze is due to the compromising effect on locomotion causing an increase in time spent immobile on the open arm of the maze rather than an increase in explorative behaviour. This compromising effect of MPEP on locomotor activities at 2.5 mg/kg was also recorded in the open field (Figure 5A). On the other hand, MPEP at 5 mg/kg compromised locomotion of ethanol withdrawn rats when tested in the EPM. Thus, the significant effect of this dose in reversing withdrawal anxiety observed in the EPM is debatable. MPEP at 10 and 30 mg/kg had neither ameliorating nor compromising effects on ethanol withdrawal induced hypolocomotion. Despite lack of significant compromising effects of MPEP dosages on the locomotion (Figures 3 and 6B), some reduction in the locomotion of ethanol naïve rats at 20 and 30 mg/kg MPEP was observed, which is consistent with previous reports with different behavioural tests [42,43]. Previous studies have reported a high abundance of mGluR5 in the nucleus accumbens and striatum [44,45], which has been heavily associated in mediating the motor effects of psychostimulants in rodents [43,46,47]. Thus, the blockade of mGluR5 in these sites could be responsible for the effects observed in the locomotion.

## Conclusions

Earlier studies and our study have reported significant anti-anxiety effects of MPEP on non-ethanol dependent rats in a number of assays in a dose range of 2.5 to 30 mg/kg [9,48]. Our study, using an elevated plus maze and an open field, reported significant effects of MPEP at 10 mg/kg in reversing ethanol withdrawal induced anxiety. Administration of MPEP at this dose also has no significant effect on the locomotor activities of ethanol naïve and ethanol withdrawn rats when tested in both behavioural paradigms. Taken together, the anxiolytic effect of 10 mg/kg MPEP in attenuating withdrawal anxiety in this study is convincing. Thus, we believe that the antagonism of mGluR5 could provide an effective pharmacological intervention in treating ethanol withdrawal induced anxiety. However, the confounding effects of MPEP at high and low doses still warrants further investigation to understand the mechanism of MPEP better.

## Competing interests

There are no competing interests among authors.

## Authors’ contributions

All authors have made substantial contributions to conception, design, acquisition, analysis and interpretation of the data, and drafting and revision of the manuscript (JK, HH, Y-TGB, ZI). All authors read and approved the final manuscript.
